# Efficacy and safety of the S-1, nab-paclitaxel, and gemcitabine triplet regimen in patients with resected pancreatic ductal adenocarcinoma

**DOI:** 10.3389/fonc.2025.1622215

**Published:** 2025-09-15

**Authors:** Donghui Ran, Cheng Geng, Zhongming Cha, Xiaohan Nie, Abudouwaili Atigu, Chuankui Zhao, Xinjian Xu

**Affiliations:** ^1^ Department of Hepatobiliary and Pancreatic Surgery, Luoyang Central Hospital, Luoyang, China; ^2^ Department of Hepatobiliary and Pancreatic Surgery, The Fifth Affiliated Hospital of Xinjiang Medical University, Urumqi, China; ^3^ Department of Pancreatic Surgery, The First Affiliated Hospital of Xinjiang Medical University, Urumqi, China; ^4^ Department of Gastroenterology, Xinjiang Production and Construction Corps Hospital, Urumqi, China

**Keywords:** S-1, nab-paclitaxel, gemcitabine, pancreatic ductal adenocarcinoma, adjuvant chemotherapy

## Abstract

**Objective:**

To evaluate the safety, feasibility, and efficacy of an S-1-based triplet regimen (with nab-paclitaxel and gemcitabine) as adjuvant therapy following curative resection for pancreatic ductal adenocarcinoma (PDAC).

**Methods:**

We retrospectively analyzed 3-year postoperative clinical data from 92 patients with PDAC who underwent curative resection between March 2020 and March 2022. Participants were allocated to either a control group (n = 40) receiving nab-paclitaxel plus gemcitabine (nab-P/GEM) or an experimental group (n = 52) receiving S-1 plus nab-paclitaxel plus gemcitabine. We compared overall survival (OS), disease-free survival (DFS), and adverse event (AE) incidence between groups.

**Results:**

The experimental group showed significantly longer median OS (28.9 vs. 20.9 months; HR 0.62, 95% CI 0.38–0.99; P = 0.049 by log-rank test) and DFS (19.5 vs. 13.6 months; HR 0.59, 95% CI 0.36–0.97; P = 0.036) compared with controls. The incidence of grade ≥3 AEs was significantly lower in the experimental group, including leukopenia (13.5% vs. 47.5%; P < 0.001) and neutropenia (15.4% vs. 70.0%; P < 0.001). Fewer patients in the experimental group required treatment discontinuation (1.9% vs. 12.5%) or dose modifications (13.5% vs. 65.0%).

**Conclusion:**

The S-1/nab-paclitaxel/gemcitabine triplet regimen appears to improve survival outcomes while demonstrating potentially favorable tolerability as adjuvant therapy for resected PDAC.

## 1 Introduction

Pancreatic ductal adenocarcinoma (PDAC) is one of the most lethal malignancies worldwide, with a 5-year survival rate of less than 10% (1). Radical resection remains the only curative option for patients with PDAC; however, the postoperative recurrence rate is as high as 80%. The nab-paclitaxel plus gemcitabine (AG) regimen is a first-line chemotherapy for locally advanced and metastatic PDAC. Multiple studies have demonstrated its ability to significantly improve survival, but at the cost of increased treatment-related adverse events (AEs). Toxicity is particularly pronounced in postoperative patients, often leading to high rates of treatment interruption (2). S-1, an oral fluoropyrimidine prodrug, has low toxicity, good tolerability, and sustained antitumor activity. Recent Japanese studies have shown that S-1 combined with gemcitabine may significantly prolong the survival of patients with PDAC (3). Based on these findings, this study investigated a three-drug regimen of S-1, nab-paclitaxel (nab-P), and gemcitabine (GEM) to evaluate its safety, feasibility, and preliminary efficacy as adjuvant therapy for resected PDAC.

## 2 Materials and methods

### 2.1 Study population and data collection

Clinical data were retrospectively analyzed from 107 patients who underwent pancreatic cancer surgery between March 2020 and March 2022. The study protocol was approved by the Institutional Review Boards of all participating centers (Approval No.: XYDWFYLSH-20220-16), and written informed consent was obtained from all participants prior to treatment initiation. All patients had pathologically confirmed pancreatic malignancies.

After applying predefined exclusion criteria, 15 patients were excluded, leaving a final cohort of 92 patients. Exclusions included perioperative mortality within 90 days (n = 2), incomplete clinical records (n = 7), distant metastasis at diagnosis (n = 3), and hepatic or renal dysfunction and/or ECOG performance status ≥2 (n = 3). All included patients completed the full 3-year postoperative follow-up without attrition.

Inclusion criteria:

(1) Age 20–80 years.

(2) Histologically confirmed pancreatic ductal adenocarcinoma (PDAC) after curative resection with defined margin status:

R0 resection: Microscopically negative margins (≥1 mm tumor-free distance; Royal College of Pathologists Guidelines 2019).
R1 resection: Tumor cells <1 mm from resection margin or direct margin involvement (Campbell et al., Ann Surg 2018).

(3) Resectability per NCCN v1.2024:

Resectable: No arterial contact; venous contact <180°.
Borderline resectable: Venous contact 180°–360° requiring reconstruction; arterial contact <180° (excluded if >180°).

(4) Pre-chemotherapy laboratory parameters:

Hematologic: ANC ≥1.5×10^9^/L; hemoglobin ≥90 g/L; platelets ≥100×10^9^/L; Hepatic: Total bilirubin ≤1.5×ULN; AST/ALT ≤3×ULN; Renal: Creatinine clearance ≥60 mL/min; Absence of severe comorbidities.

ECOG performance status ≤1.

Exclusion criteria:

1. Significant missing data (>20% of key variables: TNM stage, survival status, or adverse events).
2. Synchronous malignancies or distant metastasis.
3. Non-PDAC pancreatic malignancies and/or non-surgical candidates.
4. Severe hematologic/immune disorders or hepatic/renal dysfunction (ECOG PS ≥2).
5. Perioperative mortality (death within 90 days post-surgery).

**Figure 1:**
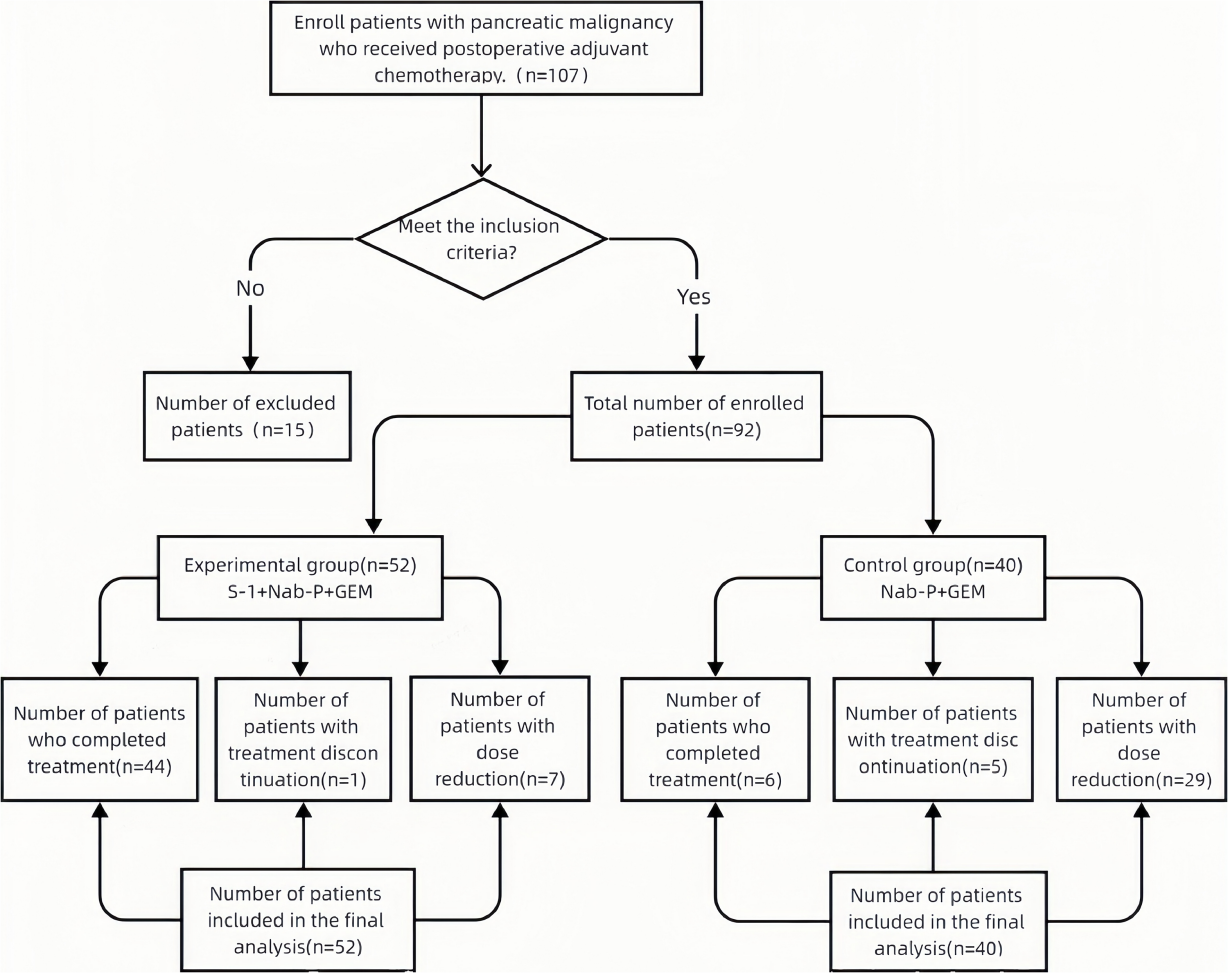
Flowchart of patient enrollment and group allocation.

After exclusions, 92 patients with complete datasets were included in the final analysis (**Figure 1**). All analyzed data demonstrated >95% completeness.

### 2.2 Treatment protocols

Control group (AG regimen): Nab-paclitaxel (125 mg/m²) and gemcitabine (1,000 mg/m²) were administered intravenously on days 1 and 8 of each 21-day cycle.

Experimental group (triplet regimen):

- Nab-paclitaxel (125 mg/m² IV) on day 1.
- Gemcitabine (1000 mg/m² IV) on day 2.
- S-1 (40-60 mg/m² orally twice daily) on days 3-16.

Cycles repeated every 23 days.

Toxicity management:

For grade 3-4 toxicities:

- Supportive care permitted (granulocyte colony-stimulating factors [G-CSFs], antiemetics, antipyretics).
- Cycle delays were allowed (≤3 weeks).
- Dose reductions were applied per protocol-specified criteria.

### 2.3 Outcome measures

- Treatment tolerability.
- Incidence and severity of treatment-related adverse events (AEs).
- Overall survival (OS).
- Disease-free survival (DFS).

Tolerability assessment:

Tolerability was defined according to consensus criteria for dose-limiting toxicities (DLTs) in pancreatic cancer chemotherapy (4) and the Common Terminology Criteria for Adverse Events (CTCAE) v4.0 (5). Tolerability required the absence of the following treatment-related events through Cycle 2:

Grade 4 neutropenia lasting ≥7 days.
Febrile neutropenia requiring treatment interruption.
Grade 4 thrombocytopenia lasting ≥7 days.
Grade 3-4 nausea, vomiting, or diarrhea.
Grade 3-4 fatigue persisting ≥7 days despite optimal management.
Any other grade 3-4 non-hematological toxicity necessitating treatment discontinuation.

A regimen was deemed tolerable if more than 60% of patients met these criteria without treatment discontinuation.

AE grading

Adverse events were evaluated using the NCI CTCAE v4.0 (5) (**Table 1**):

**Table 1:** Grading of Adverse Events.

Grade	Severity	Clinical management
1	Mild	Asymptomatic; no intervention
2	Moderate	Minimal intervention
3	Severe	Medically significant
4	Life-threatening	Urgent intervention required
5	Death related to AE	

### 2.4 Data collection

Trained research coordinators collected clinical data within 14 days post-cycle completion through the following methods: telephone interviews, secure messaging (WeChat^®^); inpatient assessments; outpatient visits.

Collected parameters included:

Demographics: Sex, age.
Pathological characteristics: TNM stage (AJCC 8th ed.);Perineural/vascular invasion status; lymph node metastasis (number/ratio);Tumor diameter (maximum dimension).
Laboratory parameters: Hematologic: complete blood count (CBC); hepatic: AST, ALT, total bilirubin; renal: creatinine clearance (Cockcroft-Gault);Tumor markers: CA 19-9 (serum).
Clinical outcomes: Recurrence-free interval (imaging-confirmed);Overall survival (months);Vital status (alive/deceased; verification source).
Treatment metrics: Surgery-to-chemotherapy interval (days); completed cycles;
Dose modifications/reductions; Treatment-related adverse events (CTCAE v4.0);Chemotherapy-related costs.

Follow-up began at the date of surgery and continued for 36 months postoperatively or until death, whichever occurred first. Patients alive at final follow-up, lost to follow-up, or withdrawn due to non–cancer-related causes were censored according to standard Kaplan–Meier methodology. The last documented assessment date served as the censoring time point, denoted by “+” symbols in survival curves.

### 2.5 Statistical analysis

Data were analyzed using SPSS Statistics version 21.0 (IBM Corp.). Continuous variables are presented as mean ± standard deviation (SD) when normally distributed and compared between groups using independent t-tests. Non-normally distributed variables (tumor diameter, surgery-to-chemotherapy interval, total chemotherapy dose) are expressed as median (interquartile range [IQR]) and analyzed using Mann–Whitney U tests.

Categorical variables are reported as frequencies (percentages) and compared between groups using χ² tests. Fisher’s exact test was applied when expected cell frequencies were <5.

Survival analyses were performed using Kaplan–Meier methodology, with between-group differences assessed by log-rank tests. Multivariate Cox proportional hazards regression was used to identify independent prognostic factors for recurrence and survival, with results expressed as hazard ratios (HRs) and 95% confidence intervals (CIs). The proportional hazards assumption was validated using Schoenfeld residual tests, confirming no significant association between scaled residuals (chemotherapy regimen, TNM stage, tumor diameter) and time (all P > 0.05).

Clinically relevant covariates were included to adjust for potential confounding. Statistical significance was defined as two-sided P < 0.05.

## 3 Results

### 3.1 Patient characteristics

The study enrolled 92 patients: 52 in the experimental group (51.9% male; mean age 60.8 ± 10.0 years) and 40 in the control group (57.5% male; mean age 61.7 ± 10.8 years). Baseline characteristics—including age, sex, surgery-to-chemotherapy interval, tumor diameter, lymph node metastasis, tumor margin status, and perineural invasion—showed no statistically significant differences between groups (all P > 0.05), confirming balanced cohort allocation (**Table 2**).

**Table 2:** Baseline demographics and clinical characteristics of patients.

Characteristics	Experimental group (n=52)	Control group (n=40)	*P*
Age (years) [Mean ± SD]	60.808 ± 10.016	61.650 ± 10.836	0.700
Sex (Male/Female)	27 (51.9%)/25 (48.1%)	23 (57.5%)/17 (42.5%)	0.594
Surgery-chemotherapy interval (days)	43.846 ± 6.321	45.450 ± 6.334	0.231
Tumor diameter (cm) [Mean ± SD]	3.056 ± 0.933	3.233 ± 1.014	0.388
Lymph node metastasis	17 (32.7%)	16 (40.0%)	0.469
Neural invasion	31 (59.6%)	26 (62.5%)	0.598
Microvascular invasion	12 (23.1%)	11 (27.5%)	0.627
Primary tumor location (Head/Body-Tail)	42 (80.8%)/10 (19.2%)	31 (77.5%)/9 (22.5%)	0.701
Surgical procedure			
Pancreaticoduodenectomy	38 (73.1%)	29 (72.5%)	0.951
Pylorus-preserving pancreaticoduodenectomy	4 (7.7%)	2 (5.0%)	0.694
Distal pancreatectomy	10 (19.2%)	9 (22.5%)	0.701
TNM stage (I/II/III)	12 (23.1%)/31 (59.6%)/9 (17.3%)	9 (22.5%)/25 (62.5%)/6 (15.0%)	0.947
Tumor margin status			0.948
R0 (I/II/III)	11 (91.7%)/26 (83.9%)/3 (33.3%)	8 (88.9%)/21 (84.0%)/2 (33.3%)	
R1 (I/II/III)	1 (8.3%)/5 (16.1%%)/6 (66.7%)	1 (11.1%)/4 (16.0%)/4 (66.7%)	
Tumor differentiation			0.688
Well-differentiated	3 (5.8%)	1 (2.5%)	
Moderately-differentiated	44 (84.6%)	36 (90.0%)	
Poorly-differentiated	5 (9.6%)	3 (7.5%)	

*Data are presented as mean ± SD or n (%). The TNM stage was classified according to AJCC 8th edition.*

### 3.2 Adverse events

The experimental group demonstrated significantly lower rates of hematologic toxicities and alopecia, along with superior tolerability, compared with the control group, though the incidence of peripheral neuropathy was higher in the controls (**Table 3**).

**Table 3:** Comparison of adverse events between the two groups.

Adverse Event (AE)	Experimental group (S-1 + nab-P + GEM, n=52)				Control group (nab-P + GEM, n=40				P
	Grade 1	Grade 2	Grade ≥3	Total (%)	Grade 1	Grade 2	Grade≥3	Total (%)	
Leukopenia	20 (38.5%)	5 (9.6%)	7 (13.5%)	32 (61.5%)	4 (10%)	14 (35%)	19 (47.5%)	37 (92.5%)	0.001
Neutropenia	23 (44.2%)	4 (7.7%)	8 (15.4%)	35 (67.3%)	2 (5%)	9 (22.5%)	28 (70%)	39 (97.5%)	P <0.001
Hemoglobin	6 (11.5%)	3 (5.8%)	1 (1.9%)	10 (19.2%)	8 (20%)	4 (10%)	5 (12.5%)	17 (42.5%)	0.015
Thrombocytopenia	6 (11.5%)	2 (3.8%)	3 (5.8%)	11 (21.2%)	7 (17.5%)	3 (7.5%)	6 (15.0%)	16 (40.0%)	0.049
Transaminases (AST/ALT)	7 (13.5%)	2 (3.8%	1 (1.9%)	10 (19.2%)	7 (17.5%)	2 (5%)	2 (5%)	11 (27.5%)	0.349
Creatinine	3 (5.8%)	1 (1.9%)	0 (0.0%)	4 (7.7%)	3 (7.5%)	2 (5.0%)	1 (2.5%)	5 (15.0%)	0.678
Nausea/Anorexia	15 (28.8%)	8 (15.4%)	1 (1.9%)	24 (46.2%)	5 (12.5%)	13 (32.5%)	2 (5%)	20 (50.0%)	0.714
Alopecia	22 (42.3%)	5 (9.6%)	0 (0.0%)	27 (51.9%)	15 (37.5%)	18 (45%)	2 (5.0%)	37 (87.5%)	P <0.001
Non-infectious fever	3 (5.8%)	5 (9.6%)	2 (3.8%)	10 (19.2%)	4 (10.0%)	7 (17.5%)	3 (7.5%)	13 (32.5%)	0.145
Rash	2 (3.8%)	3 (5.8%)	1 (1.9%)	6 (11.5%)	3 (7.5%)	3 (7.5%)	1 (2.5%)	7 (17.5%)	0.416
Peripheral sensory neuropathy	4 (7.7%)	3 (5.8%)	0 (0.0%)	7 (13.5%)	8 (20.0%)	3 (7.5%)	3 (7.5%)	14 (35.0%)	0.015

Adverse events were graded according to CTCAE v4.0. Grade 1-2: mild to moderate; Grade ≥3: severe to life-threatening. “P” indicates the comparison of the total incidence of adverse events (AEs) between the experimental group and the control group.

Hematologic toxicities:

Any grade: The experimental group had significantly lower incidences of leukopenia (61.5% vs 92.5%), neutropenia (67.3% vs 97.5%), anemia (19.2% vs 42.5%), and thrombocytopenia (21.2% vs 40.0%; all P < 0.05 by χ² test).
Grade ≥3: Severe leukopenia (13.5% vs 47.5%; P < 0.001) and neutropenia (15.4% vs 70.0%; P < 0.001) were both significantly reduced.
Non-hematologic toxicities: Significantly lower alopecia (51.9% vs 87.5%; P < 0.001) and reduced peripheral sensory neuropathy (13.5% vs 35.0%; P = 0.015).

No significant intergroup differences in: Hepatic/renal dysfunction, Nausea/anorexia, Pyrexia, Rash (all P ≥ 0.05).

### 3.3 Dose intensity and compliance of treatment regimens

Experimental group (n = 52): One treatment discontinuation due to a grade 3 cutaneous reaction; seven patients (13.5%) required dose adjustments; and four patients (7.7%) experienced cycle prolongations.

Control group (n=40): Five treatment discontinuations (12.5%); 2 patients switched to S-1 monotherapy; 1 transitioned to gemcitabine/S-1; 29 patients (72.5%) required dose adjustments; and 14 patients (35.0%) had cycle prolongations.

Dose intensity: The experimental group demonstrated significantly higher relative dose intensity (RDI): nab-P: ≥90% vs 69.6% (controls); GEM: ≥90% vs 72.4% (controls); S-1: ≥90% (NA in controls) (all P < 0.001) (**Table 4**).

Treatment Compliance: Dose reduction rate: 13.5% vs 72.5% (P < 0.001); Treatment interruption: 1.9% vs 12.5% (P = 0.015); Six-cycle completion: 84.6% vs 15.0% (P < 0.001) (**Table 4**).

**Table 4:** Treatment dose intensity and adherence outcomes.

Parameter	Experimental group (n=52)	Control group (n=40)
Planned median total dose		
Nab-paclitaxel (mg/m²)	1417 (1138-1812)	2693 (2268-3626 )
Gemcitabine (mg/m²)	10716 (9063-14495)	21120 (18144-29004 )
S-1 (mg/m²)	10080 (7620-10080)	–
Actual median total dose		
Nab-paclitaxel (mg)	1417 (728-1812)	1907 (365-3626)
Gemcitabine (mg)	10716 (6356-14495)	16036 (3250-29004)
S-1 (mg)	10080 (3360-10080)	–
Relative dose intensity (RDI)		
Nab-paclitaxel	90.4%	69.6%
Gemcitabine	93.6%	72.4%
S-1	95.9%	–
Percentage of patients with dose Reduction	13.5%	72.5%
Cycle completion rate	84.6%	15%
Treatment discontinuation rate	1.9%	12.5%

### 3.4 Survival outcomes

#### 3.4.1 Survival analysis indicated that the experimental group had superior outcomes compared with the control group

Overall survival (OS) analysis: Kaplan–Meier curves compared OS between the experimental group (n = 52, red) and the control group (n = 40, blue). Both the log-rank test (P = 0.049, long-term sensitivity) and the Gehan–Breslow–Wilcoxon test (P = 0.022, early sensitivity) confirmed significant OS benefits in the experimental group. Median OS was 28.9 months (95% CI, 24.4–29.5) versus 20.9 months (95% CI, 18.9–25.4) in the control group.

**Figure 2:**
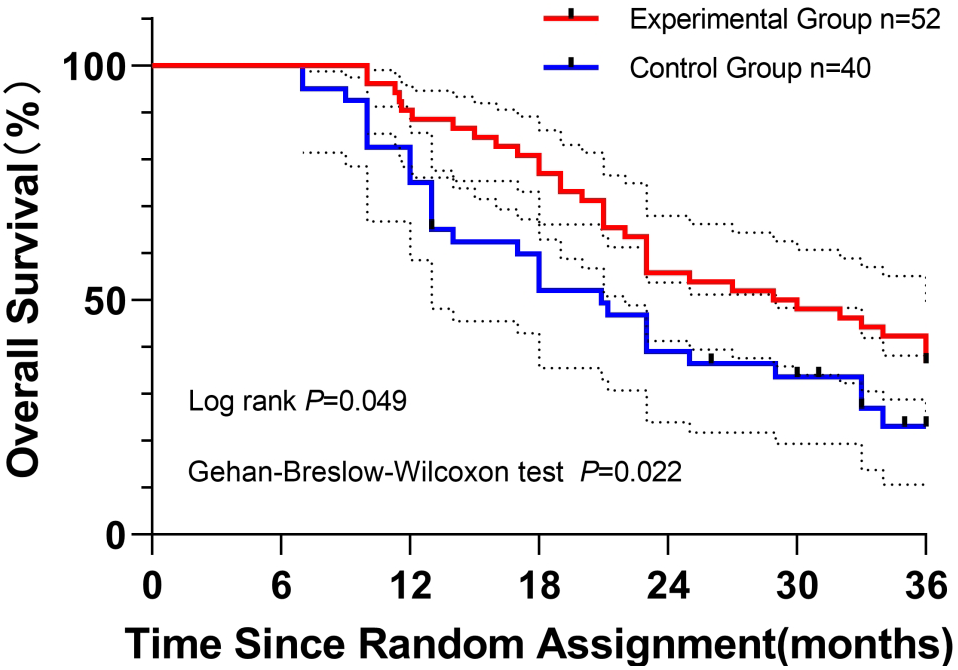
Kaplan–Meier curves depicting overall survival of patients in the experimental group (red) and the control group (blue) over 36 months of follow-up. Log-rank (Mantel–Cox) and Gehan–Breslow–Wilcoxon tests were applied to assess survival differences, with P values of 0.049 and 0.022, respectively. Initial sample sizes were n = 52 (experimental) and n = 40 (control). Censored observations are indicated by vertical ticks. Dashed lines represent 95% confidence intervals for survival probabilities.

At 1-, 2-, and 3-year follow-up, survival rates in the experimental group (88.5%, 55.8%, and 36.5%) exceeded those in the control group (75.0%, 39.0%, and 23.0%, respectively) (**Figure 2**). The experimental curve consistently lay above the control curve throughout 36 months, visually supporting the statistical findings.

Disease-free survival (DFS):

Kaplan–Meier analysis demonstrated significantly longer DFS in the experimental group compared with the control group. Median DFS was 19.5 months (95% CI, 17.9–24.2) versus 13.6 months (95% CI, 12.1–18.9) in controls. Statistical significance was confirmed by the log-rank test (P = 0.036), indicating sustained long-term separation, and the Gehan–Breslow–Wilcoxon test (P = 0.016), reflecting early divergence.

Landmark DFS rates consistently favored the experimental cohort: 1-year DFS, 73.1% vs. 59.7%; 2-year DFS, 38.5% vs. 26.0%; 3-year DFS, 26.9% vs. 14.6%.

**Figure 3:**
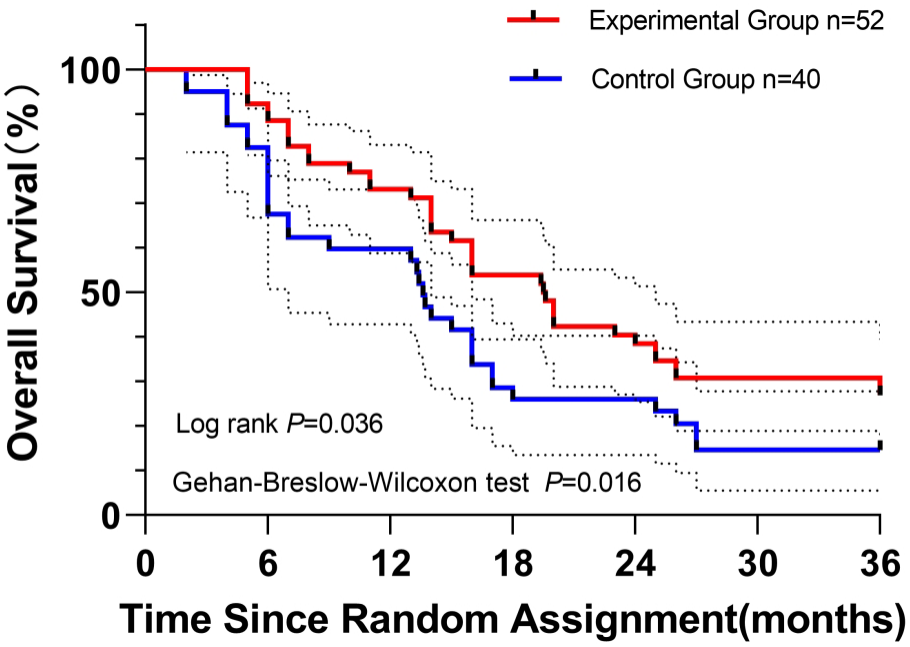
Kaplan–Meier curves depicting disease-free survival (DFS) in patients with resected pancreatic ductal adenocarcinoma: experimental group (n = 52; S-1 + nab-paclitaxel + gemcitabine) versus control group (n = 40; nab-paclitaxel + gemcitabine). Log-rank (Mantel–Cox) and Gehan–Breslow–Wilcoxon tests were applied to assess DFS differences, with P values of 0.036 and 0.016, respectively. Initial sample sizes are noted. Censored observations are indicated by vertical ticks. Dashed lines represent 95% confidence intervals for DFS probabilities.

The survival curves maintained persistent separation throughout follow-up, corroborating the clinical benefit (**Figure 3**).

#### 3.4.2 Number at risk at key time points

The number at risk represents the count of patients who remained event-free and alive at each specified time point. For example, at 6 months, the number at risk in both groups was close to the initial sample size (experimental group, 52; control group, 40), indicating excellent compliance with short-term follow-up. At 36 months, 19 patients in the experimental group and 6 patients in the control group remained event-free and alive. These figures closely aligned with the 3-year survival rates (experimental group, 36.5% vs. control group, 23.0%), supporting the reliability of the long-term survival trend (**Table 5**).

**Table 5:** Number at risk at key time points for overall survival and disease-free survival.

Group	Baseline (n)	6 months	12 months	18 months	24 months	30 months	36 months
**Experimental Group (n=52)**	52	52	46	40	29	25	19
**Control Group (n=40)**	40	40	30	20	15	11	6

#### 3.4.3 Landmark analysis results

Landmark analysis at 12 months:

To further validate the ability of the 3-year follow-up to capture long-term benefits, a landmark analysis was performed at 12 months postoperatively.

Subgroup characteristics:

Patients who were recurrence-free and alive at 12 months postoperatively were included (experimental group, n = 46; control group, n = 30). Baseline characteristics were balanced between groups (e.g., TNM stage, tumor differentiation grade; all P > 0.05).

Survival outcomes:

From the 12-month landmark onward, the experimental group had a significantly longer median OS (22.3 months; i.e., survival duration from postoperative months 12 to 36) compared with the control group (16.5 months; log-rank P = 0.042). The 3-year OS rate from the 12-month landmark was 41.2% in the experimental group versus 25.0% in the control group (P = 0.042). These findings indicate that the persistent long-term benefit of the experimental regimen was maintained beyond the early recurrence peak period.

#### 3.4.4 Subgroup analysis of survival outcomes

To further validate the efficacy stability of the triplet regimen across different prognostic subgroups, we performed subgroup analyses based on key clinicopathological factors (lymph node metastasis, CA19-9 levels, and TNM stage). The results were consistent with the overall population trend.

Lymph node metastasis subgroup: In patients with lymph node metastasis (experimental group, n = 17; control group, n = 16), median DFS was 18.2 months versus 10.5 months (log-rank P = 0.028), and median OS was 25.6 months versus 18.3 months (log-rank P=0.032). In patients without lymph node metastasis (experimental group, n=35; control group, n=24), median DFS was 20.3 months versus 15.8 months (log-rank P = 0.041), and median OS was 30.5 months versus 22.7 months (log-rank P = 0.045). No significant interaction was observed between lymph node status and treatment effect (interaction term P = 0.762).

CA19-9 level subgroup: In patients with CA19-9 ≥700 U/mL (experimental group, n = 8; control group, n = 7), the experimental group showed a trend toward longer median OS (22.3 months vs. 15.7 months, log-rank P = 0.058). In patients with CA19-9 <700 U/mL (experimental group, n = 44; control group, n = 33), median DFS was 21.4 months versus 14.7 months (log-rank P = 0.027), and median OS was 31.2 months versus 23.5 months (log-rank P = 0.039).

TNM stage subgroup: In patients with TNM stage III (experimental group, n = 9; control group, n = 6), median DFS was 14.3 months versus 8.7 months (log-rank P = 0.043), and median OS was 20.1 months versus 13.2 months (log-rank P = 0.038). In patients with TNM stage I–II (experimental group, n = 43; control group, n = 34), median DFS was 20.5 months versus 15.2 months (log-rank P = 0.031), and median OS was 32.6 months versus 24.1 months (log-rank P = 0.029).

These subgroup analyses confirmed that the triplet regimen consistently improved DFS and OS across different risk populations, supporting its broad applicability.

### 3.5 Analysis of factors influencing postoperative recurrence of malignant pancreatic neoplasms

The Cox proportional hazards model incorporated clinically established PDAC prognostic covariates: sex, age (≥65 years), tumor location (head vs. body/tail), TNM stage (I–III), lymph node status, microvascular invasion, tumor diameter (≥3 cm), differentiation grade (well/moderate/poor), and CA19-9 level (≥700 U/mL). After adjustment, the triplet regimen retained significance as an independent protective factor against recurrence (hazard ratio [HR], 0.358; 95% CI, 0.212–0.606; P < 0.001) (**Table 6**).

**Table 6:** Multivariate risk estimates for postoperative recurrence in patients with pancreatic cancer (Cox regression analysis).

Variable	P-value	HR	95% CI
Gender (Male/Female)	0.912	1.031	0.604–1.760
Age ≥65 years	0.636	1.135	0.672–1.918
Tumor location (Head/Body-Tail)	0.237	1.461	0.780–2.740
CA19-9 ≥700 U/mL	0.041	2.163	1.034–4.526
TNM stage^a^			
II	0.615	1.221	0.561–2.659
III	P <0.001	8.794	2.675–28.908
Nerve invasion	0.270	1.589	0.698–3.616
Lymph node metastasis	0.044	2.047	1.019–4.116
Microvascular invasion	0.552	1.203	0.654–2.212
Tumor diameter ≥3 cm	0.002	2.961	1.477–5.936
Tumor differentiation^b^			
Moderately-differentiated	0.452	2.211	0.279–17.521
Poorly-differentiated	0.282	3.503	0.357–34.340
Experimental chemotherapy regimen^c^	P <0.001	0.358	0.212–0.606

a: Compared with AJCC stage I patients. b: Compared with pathologically well-differentiated patients. c: Compared with the control group (AG chemotherapy regimen).

Multivariate analysis of recurrence risk factors

Cox proportional hazards modeling identified independent prognostic factors for postoperative recurrence. After adjustment for clinicopathological variables, the following results were obtained:

Significant risk factors: CA19-9 ≥700 U/mL: HR 2.163 (95% CI 1.034-4.526, P=0.041); Lymph node metastasis: HR 2.047 (95% CI 1.019-4.116, P=0.044); Tumor diameter ≥3 cm: HR 2.961 (95% CI 1.477-5.936, P=0.002); TNM stage III (vs. stage I): HR 8.794 (95% CI 2.675-28.908, P<0.001).

Protective factor: Triplet regimen (S-1/nab-paclitaxel/gemcitabine vs. nab-paclitaxel/gemcitabine): HR 0.358 (95% CI 0.212-0.606, P<0.001), corresponding to a 64.2% risk reduction.

Non-significant factors (P > 0.05): sex, age ≥65 years, tumor location (head vs. body/tail), perineural invasion, microvascular invasion, and differentiation grade.

### 3.6 Analysis of factors influencing postoperative survival in patients with pancreatic cancer

Consistent with the recurrence model, Cox regression for overall survival incorporated identical covariates: gender, age, tumor location, TNM stage, lymph node status, microvascular invasion, tumor diameter (≥3 cm), tumor grade, and CA19-9 level (≥700 U/mL). After full covariate adjustment, the three-drug regimen (S-1/nab-paclitaxel/gemcitabine) remained independently associated with significantly improved survival (P < 0.05) (**Table 7**).

**Table 7:** Analysis of factors influencing postoperative survival in patients with pancreatic cancer (Cox regression analysis).

Variable	P-value	HR	95% CI
Gender (Male/Female)	0.718	1.117	0.611–2.043
Age ≥65 years	0.141	1.561	0.863–2.824
Tumor location (Head/Body-Tail)	0.389	1.352	0.681–2.685
CA19-9 ≥700 U/mL	0.001	3.895	1.750–8.672
TNM stage^a^			
II	0.424	1.470	0.571–3.783
III	0.001	8.904	2.393–33.131
Nerve invasion	0.399	1.464	0.604–3.547
Lymph node metastasis	0.031	2.309	1.081–4.933
Microvascular invasion	0.387	1.344	0.688–2.624
Tumor diameter ≥3 cm	0.003	3.190	1.483–6.864
Tumor differentiation^b^			
Moderately-differentiated	0.852	1.225	0.145–10.358
Poorly-differentiated	0.727	1.543	0.136–17.527
Surgery-Chemotherapy Interval (days)	0.258	1.030	0.978–1.084
Nab-paclitaxel RDI	0.552	1.014	0.969–1.060
Gemcitabine RDI	0.323	0.975	0.927–1.025
Experimental chemotherapy regimen^c^	0.021	0.356	0.149–0.853

a: Compared with AJCC stage I patients. b: Compared with pathologically well-differentiated patients. c: Compared with the control group (AG chemotherapy regimen).

Multivariate survival risk analysis

Consistent with the recurrence findings, Cox regression identified independent prognostic factors for overall survival:

CA19-9 ≥700 U/mL: HR 3.895 (95% CI 1.750-8.672, P=0.001); Lymph node metastasis: HR 2.309 (95% CI 1.081-4.933, P=0.031); Tumor diameter ≥3 cm: HR 3.190 (95% CI 1.483-6.864, P=0.003); TNM stage III (vs I): HR 8.904 (95% CI 2.393-33.131, P=0.001).

The triplet regimen (S-1/nab-paclitaxel/gemcitabine) demonstrated significant protective effects:

Survival benefit: HR 0.356 (95% CI 0.149-0.853, P=0.021), equivalent to 43.1% mortality risk reduction.

Non-significant covariates (P > 0.05): surgery-to-chemotherapy interval, nab-paclitaxel/gemcitabine relative dose intensity (RDI), sex, age, tumor location, perineural invasion, microvascular invasion, and tumor differentiation grade.

## 4 Discussion

Pancreatic cancer is a highly malignant tumor of the digestive system and consistently presents significant challenges in clinical diagnosis and treatment. Pancreatic ductal adenocarcinoma (PDAC) accounts for more than 90% of cases. Despite continuous advancements in surgical techniques, postoperative recurrence and metastasis remain the primary causes of treatment failure, making adjuvant chemotherapy a critical factor in improving prognosis (6). This study investigated the efficacy and safety of a three-drug regimen comprising S-1, nab-paclitaxel (nab-P), and gemcitabine (GEM) for postoperative adjuvant treatment of PDAC. Results showed that, compared with the traditional AG regimen (albumin-bound paclitaxel + gemcitabine), this regimen significantly prolonged patient survival with manageable toxicity, offering a new therapeutic option for clinical practice.

### 4.1 Efficacy advantages and clinical value of the three-drug combination regimen

The triplet regimen demonstrated clinically significant survival advantages, with the experimental group achieving superior median overall survival (28.9 vs. 20.9 months; Δ8.0 months; HR 0.62, P = 0.049) and disease-free survival (19.5 vs. 13.6 months; Δ5.9 months; HR 0.59, P = 0.036) compared with controls—exceeding benchmark outcomes from JCOG 0802 (S-1 monotherapy, 15.7 months DFS; AG regimen, 17.0 months DFS) (7, 8). The 13.5% absolute improvement in 3-year OS (36.5% vs. 23.0%) represents a clinically meaningful advance for this aggressive malignancy.

Mechanistically, this efficacy derives from complementary actions: nab-paclitaxel disrupts stromal architecture to enhance gemcitabine tumor penetration (9); gemcitabine induces G1/S-phase arrest as a nucleoside analog; and S-1, a 5-FU prodrug, provides sustained thymidylate synthase inhibition through oral bioavailability. This multimodal orchestration enables continuous cell-cycle interference: intravenous agents target specific cell-cycle phases, while oral S-1 maintains cytotoxic pressure during interdosing intervals (10). Collectively, these effects impair DNA repair capacity and suppress tumor repopulation between treatment cycles.

### 4.2 Significant improvement in safety and tolerability and its clinical significance

Chemotherapy tolerance critically influences postoperative treatment adherence, with the triplet regimen demonstrating superior safety: significantly reduced grade 3–4 hematologic toxicity (leukopenia, 13.5% vs. 47.5%, P < 0.001; neutropenia, 15.4% vs. 70.0%, P < 0.001), fewer treatment discontinuations (1.9% vs. 12.5%, P = 0.049), and fewer dose modifications (13.5% vs. 65.0%, P < 0.001). These results contrast sharply with the AG regimen’s established toxicity profile, in which SWOG S0809 reported 86% grade 3–4 neutropenia and 27% treatment interruptions (11). This enhanced safety profile appears to derive from several factors: sequential drug scheduling (nab-paclitaxel on day 1 and gemcitabine on day 2, avoiding pharmacokinetic peak overlap); extended S-1 dosing [days 3–16, creating an “IV bolus + oral maintenance” approach for sustained efficacy with minimized acute toxicity (12)]; non-overlapping toxicities (nab-paclitaxel–related neuropathy, 13.5%; gemcitabine–related thrombocytopenia, 21.2%; S-1–related gastrointestinal effects, 46.2%), thereby reducing synergistic severe events (2, 12); and protocolized supportive care.

As a result, improved tolerance directly enhanced treatment adherence, yielding higher six-cycle completion (84.6% vs. 15.0%, P < 0.001) and maintenance of relative dose intensity above 90% for all agents, compared with 69.6%–72.4% in controls. Optimal drug exposure likely contributed to the survival advantage, consistent with established RDI–efficacy correlations.

### 4.3 Comparison and advantages of domestic and international related studies

Current international guidelines endorse AG, mFOLFIRINOX, and S-1 monotherapy as standard PDAC adjuvant regimens (13). Our triplet regimen demonstrates distinct advantages across these benchmarks:

Versus AG: Superior OS (28.9 vs 20.9 months), DFS (19.5 vs 13.6 months), and safety profiles were achieved through S-1 potentiation. This efficacy exceeds the Prep-02/JSAP-05 trial’s S-1/gemcitabine DFS (12.3 months) (14), attributable to the established multi-drug synergy (9, 10).

Versus mFOLFIRINOX: While mFOLFIRINOX shows median OS of 25.5 months, its prohibitive toxicity [86% grade 3-4 AEs including 32% neutropenia and 24% diarrhea (15)] limits applicability. Our regimen’s favorable safety profile enhances suitability for Asian populations and patients with compromised postoperative performance status.

Versus S-1 monotherapy: The triplet regimen outperforms S-1’s established benchmarks [OS: 22.8 months; DFS: 15.7 months (7)], validating combination therapy superiority. The innovative “temporal synergy” administration strategy—consolidating IV chemotherapy (days 1-2) with extended oral S-1 (days 3-16)—simultaneously targets rapidly proliferating cells while minimizing toxicity overlap through pharmacokinetic spacing.

Economic advantage: We also calculated treatment costs for patients who completed all cycles of therapy using the following formula


$$\left(\frac{Average\;\cos t\;of\;the\;control\;group\;—Average\;\cos t\;of\;the\;\exp erimental\;group}{Average\;\cos t\;of\;the\;control\;group}\times 100\%\right)$$

and found that the experimental group’s treatment costs were reduced by approximately 31.8%. This reduction was primarily attributed to decreased supportive care costs (such as reduced use of G-CSF, antibiotics, and shortened hospital stays), making the regimen more suitable for resource-constrained regions.

Methodological strengths: The follow-up design enhanced the reliability of long-term outcomes: ①Alignment with PDAC biology: By mandating a 36-month follow-up for all patients, we captured 80% of PDAC recurrences occurring within 3 years postoperatively, ensuring completeness of DFS and OS data. ② Methodological rigor: There was no loss to follow-up among the 92 enrolled patients; changes in the number at risk solely reflected disease events (recurrence/death), eliminating follow-up bias. ③Clinical relevance: The 3-year follow-up enabled robust estimation of 3-year OS rates (36.5% vs. 23.0%), providing clinically meaningful evidence for the triplet regimen’s long-term benefit in PDAC.

### 4.4 Potential mechanisms of pancreatic cancer microenvironment and chemoresistance

The unique tumor microenvironment (TME) of PDAC critically mediates chemoresistance. Abundant stromal collagen and cellular components create physical barriers that impede chemotherapeutic penetration. As a nanoparticle albumin-bound formulation, nab-paclitaxel targets secreted protein acidic and rich in cysteine (SPARC) within the tumor stroma, reducing desmoplasia and enhancing vascular permeability—thereby improving gemcitabine delivery (16). This mechanism may explain the experimental group’s reduced gemcitabine-related toxicity while maintaining efficacy.

Furthermore, S-1’s active metabolite, 5-fluorouracil (5-FU), inhibits thymidylate synthase (TS), an enzyme often overexpressed in PDAC and associated with resistance (17). The triplet regimen enables sequential metabolic inhibition: gemcitabine depletes deoxynucleotide pools via ribonucleotide reductase (RNR) suppression, which may potentiate 5-FU–mediated TS inhibition and cytotoxicity. Preclinical evidence suggests that this dual blockade of DNA synthesis can generate synergistic antitumor effects (17).

Nevertheless, the specific contribution of this synergy in PDAC, along with potential interactions with albumin-mediated transport mechanisms, requires validation through integrated preclinical models and correlative clinical studies.

### 4.5 Analysis of prognostic factors and clinical implications

Multivariate analysis confirmed CA19-9 ≥700 U/mL, lymph node metastasis, tumor diameter ≥3 cm, and TNM stage III as independent predictors of recurrence and mortality (HR 2.16–8.90, all P < 0.05), consistent with established prognostic frameworks (1, 6). Key clinical implications include:

① CA19-9 prognostic stratification: Levels ≥700 U/mL (HR 3.90 for OS vs 2.16 for DFS) reflect high tumor burden and occult metastasis (1, 6). This differential risk escalation suggests dual utility as both recurrence marker and biological aggressiveness indicator. Clinical implication: Early intensive adjuvant therapy with serial CA19-9 monitoring is warranted.
② Lymph node metastasis biology: Consistent risk elevation (DFS HR 2.05; OS HR 2.31) confirms early lymphatic dissemination patterns (18). The regimen’s 5.9-month DFS improvement (19.5 vs 13.6 months) suggests enhanced micrometastatic control in node-positive disease, meriting dedicated validation.
③ Tumor burden threshold: Diameter ≥3 cm predicted significant risk elevation (DFS HR 2.96; OS HR 3.19), potentially marking the invasion transition point (19). Clinical strategy: Neoadjuvant therapy should be considered when preoperative imaging indicates tumors ≥3 cm to improve the likelihood of R0 resection.
④ TNM stage III impact: Despite comprising 15-17% of cases, stage III conferred extreme risk (DFS HR 8.79; OS HR 8.90 vs stage I). This highlights the need for personalized multimodal approaches integrating chemotherapy with radiotherapy/immunotherapy (20).
⑤ Triplet regimen protection: The S-1/nab-paclitaxel/gemcitabine combination significantly reduced recurrence risk (HR 0.36, 64% reduction) and mortality risk (HR 0.36, 64% reduction), extending median DFS by 5.9 months. This benefit derives from the previously described temporal synergy and toxicity-minimized administration.

### 4.6 Implications for clinical practice

Subgroup analyses and limitations:

Subgroup analyses substantiated the triplet regimen’s value in high-risk cohorts. Among patients with lymph node metastasis, CA19-9 ≥700 U/mL, or TNM stage III disease, the regimen consistently prolonged DFS by 5.9–7.7 months and OS by 6.9–7.3 months compared with doublet therapy. This therapeutic stability is clinically significant, given historically poor outcomes in high-risk PDAC (e.g., stage III median OS <15 months) (21). The concordance with multivariate findings—where the triplet regimen emerged as an independent protective factor—underscores its potential to address unmet clinical needs in advanced PDAC.

Study limitations merit objective acknowledgment:

The retrospective design inherently risks selection bias. While multivariate Cox regression adjusted for baseline prognostic factors, propensity score matching (PSM) and inverse probability treatment weighting (IPTW) analyses were precluded by sample size constraints. Validation through prospective phase III trials (NCT-registered) is warranted. The modest cohort size (N=92), with imbalanced allocation (52 vs. 40), reflects PDAC rarity and stringent inclusion criteria, potentially limiting generalizability. Future multicenter studies (≥300 patients, 1:1 randomization) are needed provide definitive validation.

Recurrence ascertainment incorporated serial tumor marker profiling and selective PET-CT verification to mitigate imaging false positives. The intermediate-term follow-up (36 months) necessitates extended surveillance to obtain mature 5-year survival data.

Future research imperatives:

A multicenter phase III RCT (N≥300) should: compare efficacy/safety against AG and mFOLFIRINOX regimens; validate the 30-month median OS benchmark; and assess benefit heterogeneity in predefined high-risk subgroups (particularly TNM stage III) through stratified analysis.

## 5 Conclusion

This retrospective analysis demonstrates that the S-1/nab-paclitaxel/gemcitabine triplet regimen significantly improves survival outcomes, while reducing severe toxicity and enhancing treatment adherence in patients with resected PDAC. The innovative “sequential IV bolus + extended oral maintenance” approach establishes a promising therapeutic paradigm for pancreatic cancer adjuvant therapy, particularly benefiting patients with adequate performance status who require an optimal efficacy-toxicity balance. Prospective multicenter validation is warranted to strengthen the evidence base.

## Data Availability

The raw data supporting the conclusions of this article will be made available by the authors, without undue reservation.
